# Quantum Drugs (Q-Drugs): A New Discovery and Taboo Breaking Approach; Producing Carbon Quantum Dots from Drug Molecules

**DOI:** 10.3390/ph18060767

**Published:** 2025-05-22

**Authors:** Gamze Camlik, Besa Bilakaya, Gökçe Karaotmarlı Güven, Esra Küpeli Akkol, Zelihagül Degim, Eduardo Sobarzo-Sánchez, Ismail Tuncer Degim

**Affiliations:** 1 Department of Pharmaceutical Technology, Faculty of Pharmacy, Biruni University, Istanbul 34015, Türkiye; gcamlik@biruni.edu.tr (G.C.); bbilakaya@biruni.edu.tr (B.B.); gguven@biruni.edu.tr (G.K.G.); zdegim@biruni.edu.tr (Z.D.); 2 Biruni University Research Center (B@MER), Biruni University, Istanbul 34015, Türkiye; 3 Department of Pharmacognosy, Faculty of Pharmacy, Gazi University, Ankara 06330, Türkiye; esrak@gazi.edu.tr; 4 Centro de Investigación en Ingeniería de Materiales-CIIMAT, Facultad de Medicina y Ciencias de la Salud, Universidad Central de Chile, Santiago 8330507, Chile

**Keywords:** q-drugs, quantum dots, production, microwave synthesis, carbon quantum dots, characterizations

## Abstract

**Background/Objectives:** Carbon quantum dots (CQDs) are carbon-based structures with particle sizes ranging from 1 to 10 nm. They can be prepared using various carbon sources, including those doped with heteroatoms. CQDs exhibit unique optoelectronic properties, high photostability, low toxicity, and exceptional biocompatibility. It was aimed to produce CQDs from active pharmaceutical ingredients (APIs). **Methods:** This study introduces a novel class of CQDs synthesized directly from APIs, which we term “Quantum Drugs” (Q-Drugs). We present several APIs alongside detailed methods for Q-Drug synthesis and characterization. We describe the necessary structural properties for forming Q-Drugs and provide the values for particle size, polydispersity index, and zeta potential that were obtained from various drug molecules. **Results:** The particle sizes were determined with the size of 7.360 ± 0.030 nm and 10.000 ± 0.022 nm; polydispersity indexes of 10.500 ± 1.230 and 32.610 ± 1.401; and zeta potentials of −3.400 ± 0.054 mV and −40.000 ± 0.142 mV, respectively using different APIs. **Conclusions:** This study successfully demonstrated the synthesis and characterization of Q-Drugs, a novel class of CQD derived from APIs. The results provide valuable data on the physicochemical properties of these Q-Drugs, paving the way for further investigation into their potential applications.

## 1. Introduction

Carbon quantum dots (CQDs) were first obtained in 2004 by Xu et al., who used preparative electrophoresis to isolate them from single-walled carbon nanotubes [1]. CQDs, a class of carbon nanomaterials, show significant potential in biological applications and encompass various subtypes, including graphene quantum dots (GQDs) [2]. These hemispherical nanoparticles, composed of amorphous or crystalline carbon with dimensions of 1–10 nm, are the subject of extensive research due to their advantageous properties: high luminescence, chemical stability, water solubility, low photobleaching, biocompatibility, low cost, and low toxicity [3,4].

Compared to traditional quantum dots (QDs), CQDs offer a broader range of solutions [5]. Conventional QDs typically consist of heavy metal cores and wide-bandgap semiconductor shells, resulting in toxicity concerns. In contrast, the lower toxicity of CQDs makes them preferable for biomedical applications [6,7]. Applications for CQDs span various fields, including biological imaging, medical diagnostics, biosensors, chemical sensors, photocatalysis, and photovoltaic devices. Their ability to act as electron donors and acceptors, coupled with their capacity for chemiluminescence and electrochemiluminescence, makes them suitable candidates for optoelectronic, catalytic, and sensing applications [8,9].

CQD synthesis methods are broadly classified as top-down or bottom-up approaches. Top-down methods involve the physical or chemical breakdown of larger carbon materials into smaller CQD. Bottom-up methods synthesize CQDs from molecular precursors through processes such as carbonization [10]. The surface of CQDs typically contains functional groups such as carboxyl, hydroxyl, or amino groups; their composition varies depending on the precursor and reaction conditions, leading to differences in CQD properties [11].

CQDs surpass conventional semiconductor quantum dots due to their superior fluorescence properties, low toxicity, ease of surface functionalization, diverse precursor sources, low cost, and excellent biocompatibility [12]. Synthesis methods significantly impact UV-Vis absorption and photoluminescence; surface passivation, doping, and other post-synthesis treatments can enhance fluorescence intensity and quantum yield [13,14].

CQDs are graphitic core and shell layers saturated with hydrogen atoms and functional groups. CQDs can be modified through chemical (e.g., surface functionalization, also known as passivation or doping) and physical (e.g., core-shell architecture, composite material blending) strategies to control or tune their properties. Such modifications allow for the adjustment of photoluminescence, absorbance, and relativity. The dependence of photoluminescence upon size, edge orientation, surface and edge functionalization, doping, excitation wavelength, concentration, pH, and aggregate formation has been reported, with related theoretical evidence available in the literature [15]. Another study reported the synthesis of doped CQDs with various surface functional groups to improve size uniformity, photostability, and biocompatibility. Electrostatic and covalent surface modifications have been employed to functionalize CQD, often using diverse carbon sources and surface passivation strategies [16]. Although there are a few examples for various aims, the direct use of active substances to synthesize CQDs is not present in the literature.

In recent years, CQDs have garnered significant attention among novel nanoparticles for theranostic applications (combining diagnostic and therapeutic functionalities) in biomedicine. Their multifunctionality and low toxicity make them particularly promising for targeted therapies, diagnostics, and therapeutics [14]. A recent review comprehensively summarizes CQD synthesis, optical properties, biomedical applications, and potential challenges in the biomedical field, including proposed solutions [17].

**Figure 1:**
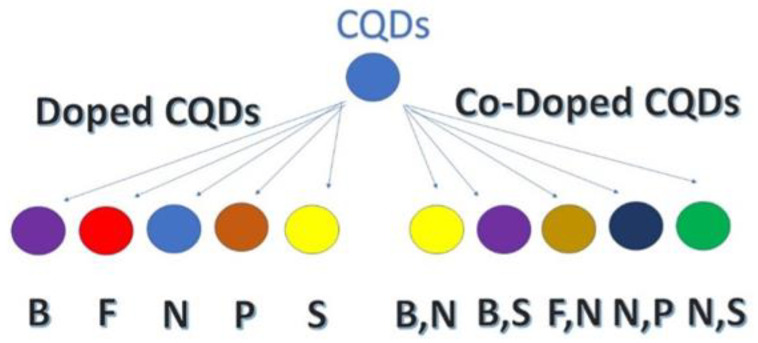
Types of CQD.

CQDs are increasingly utilized in bioanalytical applications, including in vitro and in vivo bioimaging, chemical probing, biomolecule detection and analysis, drug delivery, photoacoustic imaging, and cancer therapy [16,18]. Promising results include the observation that oral CQD administration lowered blood glucose levels [19]. CQDs can be synthesized using various methods, employing materials such as citric acid as a carbon source and nitrogen-containing dopants like l-cysteine [9,20]. Figure 1 summarizes the materials used for doping so far.

Active substances themselves can be used as both the carbon source and for the surface passivation. The discovery of CQDs was reported to be by chance, and its potential was understood later on. No flickering photoluminescence and the tunable properties of CQDs were shown [21]. The surface passivation by organic molecules like PEG stabilizes and controls the final properties of CQDs and it provides high dispersibility and excitation-dependence. They concluded that the surface functionalization of CQDs provides high dispersibility and excitation-dependence. The other group demonstrated the water-soluble CQD’s potential for catalytic applications. The dispersibility and solubility of CQDs were improved after these modifications and they observed the size-dependence of optical properties [22]. These modifications are also reported to be enriched quantum yields (QYs %). A report from the other group was a real success, showing that reductive treatments after the synthesis can enhance QYs, scaling up to 16% from 1.6% [23].

Doping and co-doping may utilize different materials, but the fundamental process involves incorporating doped or co-doped materials into the structure of the material to be carbonized. These materials confine charge carriers (electrons and holes) in a volume on the order of the particles’ quantum mechanical wavelength in the semiconductor hetero-structure. This results in CQD formation. In Q-Drugs, production of CQDs is achieved from the drug molecules: drug molecules are incorporated into the structure and present at their surface at the same time, whereas traditional drug delivery methods typically involve loading active substances onto pre-formed CQDs. This entails producing CQDs first and then using the carbon’s hydrophobic and absorbent properties to load the drug.

This study reports a significant advance in CQD synthesis. Our experimental results and a review of the literature indicated the feasibility of producing CQDs directly from drug molecules without requiring additional carbon sources or dopants. Drug molecules can function as either carbon sources or as dopants (heteroatoms). While the full implications of this discovery are yet to be determined, it has the potential to transform the field. This method facilitates the production of pharmacologically active CQDs or allows for the loading of active molecules onto the CQD surface during the synthesis process. Depending on their structures and elemental composition, drug molecules can function as a primary carbon source followed by adsorption, or as a primary heteroatom source followed by adsorption. The presence of suitable carbon atoms enables them to act as carbon sources, while the presence of available heteroatoms allows them to serve as dopants.

This discovery enables the direct synthesis of CQDs from the drug molecule itself. We have designated this approach as “Q-Drugs”. Our group has previously synthesized N-doped CQDs using citric acid (a carbon source) and l-cysteine (a nitrogen source) on litmus paper. We observed that many drugs have a high potential to yield CQDs, some even possessing nitrogen atoms in their structures. Permeation tests revealed rapid penetration of these molecules [24]. Q-Drugs may exhibit different behavior and greater efficacy than conventional nanoparticles. Nanoparticle solution behavior significantly impacts penetration, biological activity, and bioavailability [20].

**Figure 2:**
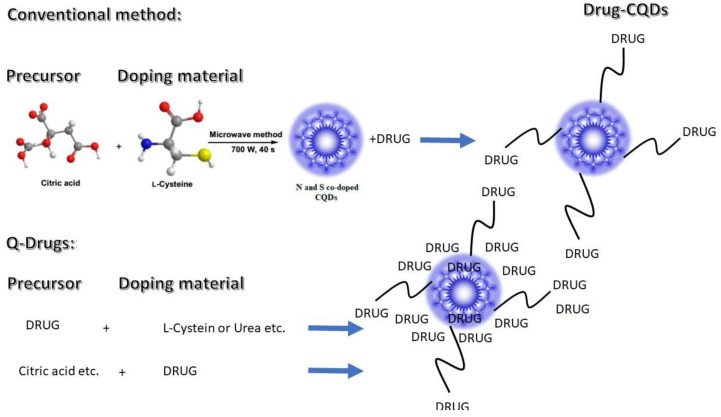
The theology and production methods of CQDs and new Q-Drugs.

Our team has successfully produced several Q-Drugs. We report, for the first time, that salicylic acid, sulfadiazine, caffeine, paracetamol, indomethacin, α-lipoic acid, hyaluronic acid, metformin, bupivacaine, lidocaine, enalapril, sertraline, gabapentin, citicoline, favipiravir, citrulline, mesalazine, and bromelain can be used to synthesize Q-Drugs. A simple, one-pot method (heating to 100–180 °C for 0–5 min, microwave irradiation for 20–25 min, and cooling) produces highly effective Q-Drugs, as reported in our recent publications [19]. Figure 2 summarizes the details of the conventional production method of CQD and new Q-Drugs.

Q-Drug synthesis offers a novel approach to producing more thermodynamically active drugs with enhanced activity and different mechanisms of action. This approach may lead to faster onset times and reduced dosages. Q-Drugs represent a potential new generation of therapeutics and may offer effective treatment strategies. Moreover, permeation tests reveal rapid penetration of drug-loaded CQDs [24].

## 2. Results

### 2.1. One-Pot Production Q-Drugs

The code, content, process parameters, appearance under UV (365 nm), and colors of Q-Drugs prepared by the microwave reactor method are given in Table 1.

### 2.2. Characterization of Q-Drugs

#### 2.2.1. Particle Size

Particle size and distribution, and zeta potential of the prepared Q-Drugs are given in Table 2.

#### 2.2.2. Stability

**Figure 3:**
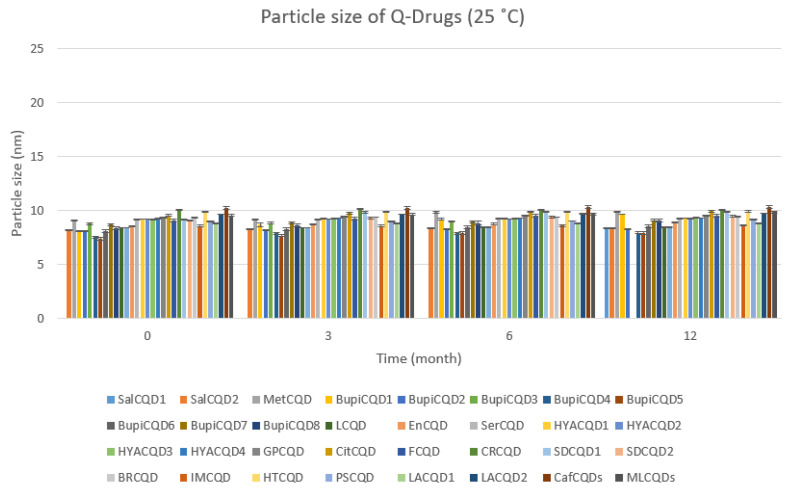
Particle size of Q-Drugs (25 °C).

**Figure 4:**
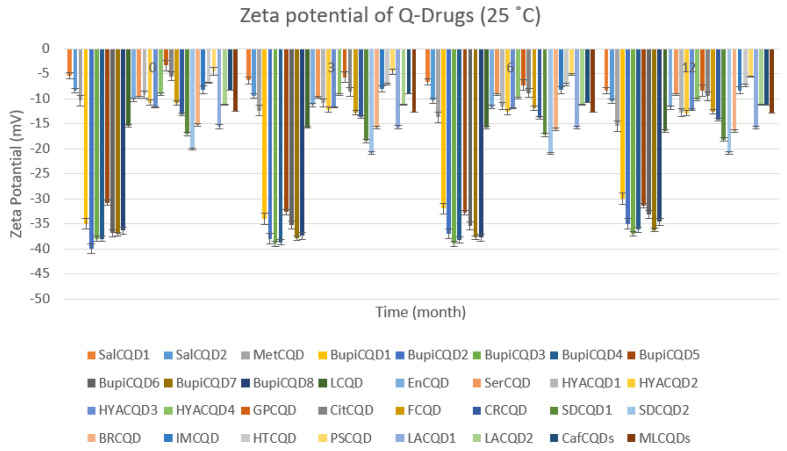
Zeta potential of Q-Drugs (25 °C).

**Figure 5:**
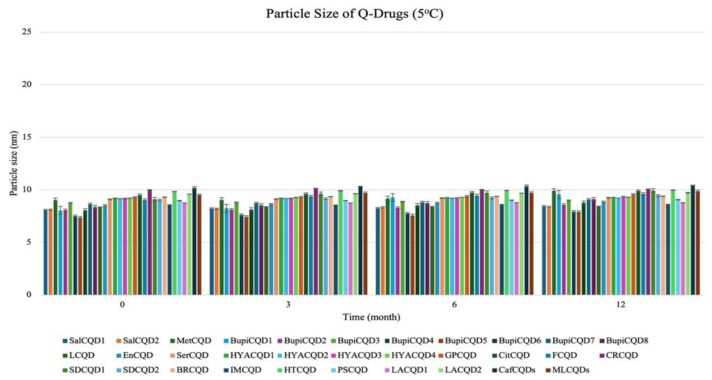
Particle size of Q-Drugs (5 °C).

**Figure 6:**
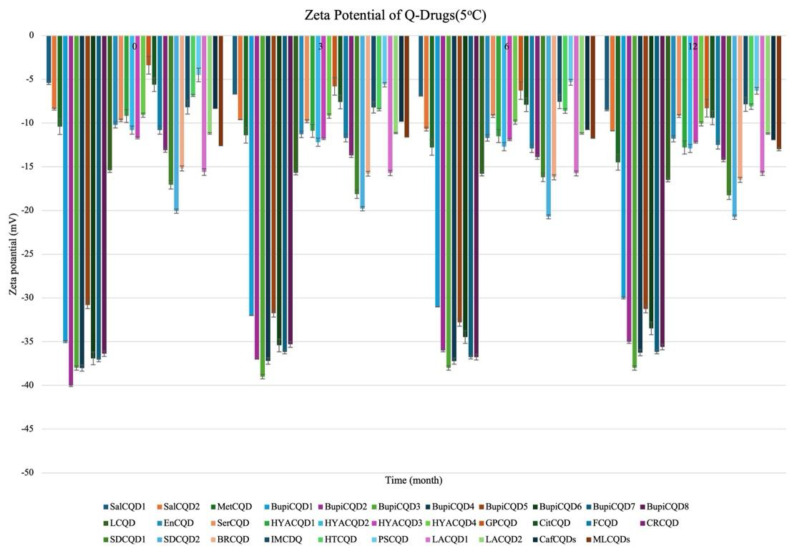
Zeta potential of Q-Drugs (5 °C).

Q-Drugs formulations were subjected to storage conditions at 5 °C and 25 °C, 60% RH, and their shelf lives were determined. The results obtained are given in Figure 3, Figure 4, Figure 5 and Figure 6. It was determined that the formulations showed a shelf life exceeding 24 months when stored in a deep freezer or refrigerator.

## 3. Discussion

In the literature, CQDs are referred to as a new class of fluorescence small carbon nanoparticles with very small particle sizes and have a variety of applications in medical fields [25]. CQDs possess quite strong and tunable fluorescence properties which enable their applications in various medical fields [26,27,28]. The synthesis and the surface properties, namely functionalization, of CQDs are reported to be important in terms of their functions and capabilities, which are made by adding organic and polymeric molecules on their surfaces [29,30].

In this study, the carbon sources or heteroatoms were chosen from drug molecules and CQDs were produced using solely drug substances reporting for the first time. 

Microwave-synthesized Q-Drugs, prepared using various active ingredients, exhibited fluorescence under UV light, displaying different colors for each composition. This observation is a key indicator of CQD formation (Table 3). Surface roughness or damage on Q-Drug particles can alter particle size, color, and brightness (Table 1). Notably, the particle sizes of the prepared Q-Drugs were below 10 nm (Table 2) [16]. The fact that Q-Drugs have a particle size below 10 nm supports the formation of carbon quantum dots.

CQDs have been reported to represent colorful photoluminescence as the excitation wavelength becomes longer, and the emission wavelength of the CQDs exhibited a continuous redshift [31]. It was later understood that nitrogen doping into CQDs was possible and affected their optical properties. The nitrogen-doped carbon dots were also found to exhibit high photoluminescence [32]. Since then, CQDs have become tunable and many efforts have been made to improve their properties through heteroatom doping [33,34]. A lot of doping strategies have been applied to produce heteroatom-doped CQDs in the literature before now. Various elements have been proposed to be used for doping even more than one since multi-atom co-doping became available. Additional atoms, such as nitrogen and boron, were incorporated into the Q-Drug structure using L-cysteine, urea, EDA, and boric acid. This resulted in a defined atomic orientation within the crystal structure, yielding bright fluorescence (blue, green, greenish-yellow, red, etc.) under UV (365 nm) illumination (Table 1) [19]. The particle sizes of the prepared Q-Drugs ranged from 7.360 ± 0.030 nm to 10.000 ± 0.022 nm, demonstrating that particle size can be modulated by varying the active substance and surface properties [35].

The PDI% values of the prepared Q-Drugs ranged from 10,500 ± 1230 to 32,610 ± 1401, indicating a relatively homogeneous distribution [36].

The zeta potential is an essential parameter reflecting the surface charge of CQDs, which influences the electrostatic forces between particles in a dispersion. Research has documented various zeta potential values for CQDs. The presence of negatively charged groups on CQD surfaces typically results in a negative zeta potential. In CQD solutions, the zeta potential varies with concentration, generally decreasing as carbon concentration rises [37]. The stability of colloidal particles depends on both the sign and magnitude of their zeta potential; higher absolute values of zeta potential typically imply greater stability, as they indicate strong repulsive forces that help prevent particle aggregation [38]. At elevated carbon concentrations, CQDs are more likely to approach each other closely, raising the probability of van der Waals and other non-covalent interactions that can lead to aggregation and particle size increase. Intermolecular forces such as van der Waals attractions, hydrogen bonding, and electrostatic interactions are critical in driving CQD aggregation [39]. Among these, van der Waals forces—weak but pervasive attraction forces between molecules—significantly impact carbon-based materials.

Zeta potential is a crucial parameter for nano formulations, influencing stability and interactions with biological systems [40,41]. The measured zeta potential for the Q-Drug formulations ranged from −3.400 ± 0.054 to −40.000 ± 0.142 in our study.

Our investigation rigorously demonstrates that the resultant CQDs exhibit significant structural and dimensional heterogeneity, distinct from the precursor drug molecules. Furthermore, a clear decoupling between the CQDs’ surface potential was observed, encompassing surface charge and functional group composition, along with their resultant luminescence properties. This intricate interplay underscores the non-trivial nature of CQD formation, highlighting a process governed by a multitude of interacting parameters.

The central significance and novel contribution of this study lie in elucidating the intricate production landscape of Q-Drugs. Our findings unequivocally demonstrate the high sensitivity of CQD characteristics to subtle variations in reaction temperature and procedural programming, often leading to unpredictable outcomes, including the complete absence of CQD formation under non-optimized conditions. The presented results are a culmination of meticulous optimization, establishing a robust yet delicate set of synthesis parameters.

This work represents the pioneering effort in leveraging actual drug molecules as direct carbon and heteroatom precursors for CQD synthesis. Our innovative approach involves the strategic placement of secondary drug molecules near the nascent CQDs during their formation. This proximity facilitates the direct and immediate integration of drug molecules into the CQD surface structure, fundamentally altering their adsorption behavior, functionalization, luminescence spectra, size distribution, and surface charge. Consequently, each Q-Drug entity exhibits a unique set of physicochemical properties dictated by the specific drug molecule employed.

The complexity inherent in this synthesis process proved to be exceptionally high, rendering accurate a priori prediction of Q-Drug characteristics virtually unattainable. Despite employing advanced computational methodologies, including artificial intelligence and neural network analysis, we were unable to establish a reliable predictive model. Therefore, this manuscript presents the initial, empirically derived observations of this novel Q-Drugs synthesis strategy. Future research endeavors will be directed towards unraveling the underlying mechanisms governing this complex self-assembly process and exploring the potential for predictive modeling.

## 4. Materials and Methods

### 4.1. Materials

Salicylic acid, sulfadiazine, caffeine, citric acid monohydrate (CAMH), urea, sodium borate (Na-Borat) boric acid, paracetamol, indomethacin and ethylenediamine (EDA), and α-lypolic acid were purchased from Sigma Aldrich (Sigma Aldrich Chemie GmbH, Taufkirchen, Germany). L-cysteine was purchased from Biobasic (20 Konrad Crescent, Markham, ON, Canada). Bupivacine and lidocaine were donated from Haver Pharma, Istanbul, Türkiye. Metformin and sertralin were donated from Sanovel, Istanbul, Türkiye. Enalapril, citicolin, and mesalazine were obtained from the market in Türkiye. GABA-Pentin was donated from Biofarma Drug Company, Istanbul, Türkiye. Favipiravir was donated from Atabay, Istanbul, Türkiye. Hyaluronic acid (HYA), citrulline, and bromelain were purchased from Life in, Proflex, and Solgar Drug Company, Istanbul, Türkiye, respectively.

### 4.2. One-Pot Production of Composite Q-Drugs

A microwave reactor (Microwave 300, Anton Paar, St. Albans, UK) was used for Q-Drug synthesis.

The contents and microwave settings for the Q-Drugs prepared are given in Table 3.

### 4.3. Characterization of Q-Drugs

Particle size distribution and zeta potential measurements of Q-Drugs were performed using an Anton Paar LiteSizer 500 (Anton Paar, St. Albans, UK). The dilutions were made prior to determination as described in the literature [42].

### 4.4. Stability

The prepared formulation was subjected to stability (at 5 °C in refrigerator and 25 °C, 60% RH) testing to determine chemical and physical stability.

### 4.5. Statistical Evaluation

Results were evaluated using the mean ± standard deviation of values obtained from the prepared formulations, with the number of replicates indicated. Statistical analysis employed a two-way ANOVA test.

## 5. Conclusions

This study demonstrates the successful microwave synthesis of Q-Drugs using drug molecules as the sole starting materials. This novel approach directly converts drug molecules into Q-Drugs, eliminating the need for conventional carbon sources or heteroatoms.

Characterization reveals that the fluorescence properties of the synthesized Q-Drugs vary significantly depending on the parent drug molecule’s structure. The resulting Q-Drugs possess a unique, nanocrystalline structure exhibiting quantum properties, which enhances their physicochemical and thermodynamic activities. Importantly, the drug molecules spontaneously adsorb onto these nanocrystals, eliminating the need for further loading.

This innovative methodology offers significant advantages. The synthesized Q-Drugs show promise for enhanced therapeutic efficacy and provide a platform for discovering additional Q-Drugs using similar synthetic strategies. This paradigm shift may facilitate the development of novel therapeutic options, offering new avenues for drug formulation and delivery across diverse medical fields.

In conclusion, the microwave synthesis of Q-Drugs directly from drug molecules significantly advances drug development. By leveraging the unique properties of these quantum-state nanocrystals, this approach has the potential to revolutionize therapeutic strategies, improving treatment efficacy and expanding patient care options. The proper Q-Drugs can be chosen from the list provided according to the need and more scientific experiments can be performed.

## Figures and Tables

**Table 1:** Appearances and fluorescence colors of Q-Drugs under UV (365 nm).

Code	Physical Appearances	Color
SalCQD1	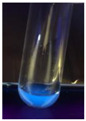	Blue
SalCQD2	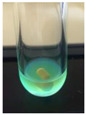	Bright Green
MetCQD	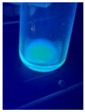	Blue-Green
BupiCQD1	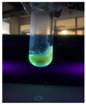	Yellowish Green
BupiCQD2	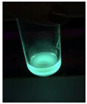	Blue
BupiCQD3	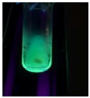	Bright Green
BupiCQD4	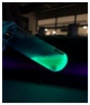	Bright Green
BupiCQD5	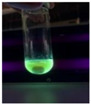	Yellowish Green
BupiCQD6	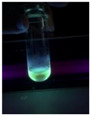	Green Blue
BupiCQD7	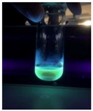	Bright Turquoise-Green
BupiCQD8	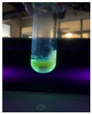	Yellowish Blue
LCQD	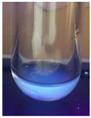	Blue
EnCQD	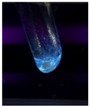	Blue
SerCQD	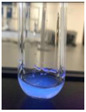	Blue
HYACQD1	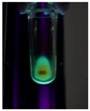	Green
HYACQD2	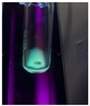	Blue
HYACQD3	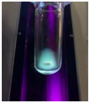	Bright Blue-Turquoise
HYACQD4	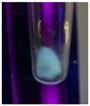	Blue
GPCQD	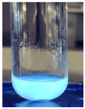	Bright Blue
CitCQD	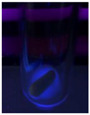	Bright Blue
FCQD	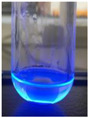	Bright Blue
CRCQD	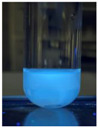	Bright Blue
SDCQD1	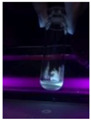	Transparent Blue
SDCQD2	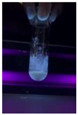	Light Transparent Blue
BRCQD	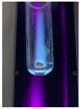	Blue
HTCQD	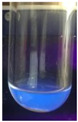	Blue
IMCQD	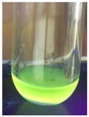	Yellowish Green
PSCQD	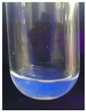	Blue
LPCQD1	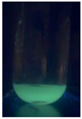	Green
LPCQD2	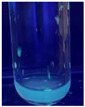	Blue
CafCQD	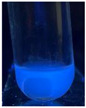	Bright Blue
MLCQD	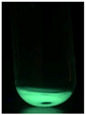	Bright Green

**Table 2:** Particle size, polydispersity index, and zeta potential measurement results of Q-Drugs after reaction.

Codes	Particle Size (nm)	Polydispersity Index % (PDI %)	Zeta Potential (mV)
SalCQD1	8.040 ± 0.031	18.050 ± 0.102	−35.000 ± 0.124
SalCQD2	8.060 ± 0.213	20.000 ± 0.102	−40.000 ± 0.142
MetCQD	8.750 ± 0.302	19.200 ± 0.102	−38.000 ± 0.109
BupiCQD1	7.510 ± 0.221	20.300 ± 0.312	−38.020 ± 0.032
BupiCQD2	7.360 ± 0.030	20.000 ± 0.102	−30.810 ± 0.035
BupiCQD3	8.050 ± 0.209	20.000 ± 0.102	−36.910 ± 0.129
BupiCQD4	8.650 ± 0.310	20.000 ± 0.102	−37.060 ± 0.042
BupiCQD5	8.360 ± 0.014	20.000 ± 0.102	−36.370 ± 0.253
BupiCQD6	8.360 ± 0.024	15.300 ± 0.012	−15.400 ± 0.032
BupiCQD7	8.520 ± 0.032	18.500 ± 0.038	−9.200 ± 0.042
BupiCQD8	9.100 ± 0.042	19.100 ± 0.016	−9.700 ± 0.023
LCQD	9.200 ± 0.012	17.500 ± 0.018	−9.200 ± 0.022
EnCQD	9.150 ± 0.122	16.700 ± 0.035	−10.800 ± 0.012
SerCQD	9.170 ± 0.045	16.900 ± 0.078	−11.700 ± 0.502
HYACQD1	9.210 ± 0.042	17.300 ± 0.033	−9.100 ± 0.102
HYACQD2	9.130 ± 0.072	18.500 ± 0.038	−9.500 ± 0.021
HYACQD3	9.290 ± 0.051	14.400 ± 0.006	−3.400 ± 0.054
HYACQD4	9.520 ± 0.151	10.500 ± 1.230	−5.600 ± 0.084
GPCQD	10.000 ± 0.022	10.700 ± 1.024	−13.100 ± 0.132
CitCQD	9.120 ± 0.042	35.500 ± 1.340	−17.060 ± 1.025
FCQD	9.040 ± 0.320	32.610 ± 1.401	−20.080 ± 1.241
CRCQD	9.310 ± 0.048	20.500 ± 0.029	−15.200 ± 0.104
SDCQD1	8.040 ± 0.031	18.050 ± 0.102	−35.000 ± 0.124
SDCQD2	8.060 ± 0.213	20.000 ± 0.102	−40.000 ± 0.142
BRCQD	8.750 ± 0.302	19.200 ± 0.102	−38.000 ± 0.109
HTCQD	9.840 ± 0.054	20.400 ± 0.121	−6.850 ± 0.110
IMCQD	8.580 ± 0.081	19.490 ± 0.550	−8.200 ± 0.781
PSCQD	8.960 ± 0.024	22.350 ± 0.025	−4.500 ± 0.301
LPCQD1	8.750 ± 0.060	18.130 ± 0.050	−15.610 ± 1.382
LPCQD2	9.600 ± 0.015	16.700 ± 1.067	−11.150 ± 0.084
CafCQD	10.190 ± 0.107	16.200 ± 1.021	−8360 ± 0.104
MLCQD	9.520 ± 0.162	17.240 ± 0.035	−12.600 ± 0.054

**Table 3:** Reaction parameters of Q-Drugs for microwave synthesis.

Code	Contents	Process/Settings
SalCQD1	0.1 g salicylic acid, 0.05 g urea 1 mL distilled water	180 °C—20 min
SalCQD2	0.1 g salicylic acid, 100 µL EDA, 2 mL distilled water	180 °C—20 min
MetCQD	0.2 g CAMH, 0.5 g metformin, 1 mL distilled water	160 °C—20 min
BupiCQD1	0.1 g Bupivacaine, 1 mL distilled water	180 °C—20 min
BupiCQD2	0.05 g Bupivacaine, 0.2 g CAMH, 2 mL distilled water	180 °C—20 min
BupiCQD3	0.03 g Bupivacaine, 0.03 g salicylic acid, 100 µL EDA, 0.03 g Na-borate, 4 mL distilled water	185 °C—20 min
BupiCQD4	0.3 g Bupivacaine, 0.03 g salicylic acid, 100 µL EDA, 4 mL distilled water	185 °C—20 min
BupiCQD5	0.3 g Bupivacaine, 0.03 g salicylic acid, 100 µL EDA, 2 mL distilled water	185 °C—20 min
BupiCQD6	0.1 g Bupivacaine, 0.03 g salicylic acid, 100 µL EDA, 2 mL distilled water	185 °C—20 min
BupiCQD7	0.1 g Bupivacaine, 0.05 g salicylic acid, 100 µL EDA, 2 mL distilled water	185 °C—20 min
BupiCQD8	0.1 g Bupivacaine, 0.1 g salicylic acid, 100 µL EDA, 2 mL distilled water	185 °C—20 min
LCQD	0.05 g Lidocaine, 0.2 g CAMH, 1 mL distilled water	160 °C—20 min
EnCQD	0.2 g Enalapril, 1 mL distilled water	160 °C—30 min
SerCQD	0.1 g Sertraline, 0.03 g urea, 1 mL distilled water	150 °C—20 min
HYACQD1	500 µL HYA solution, 0.05 g caffeine, 0.005 g L-cysteine, 0.05 g Na-Borate, 1 mL distilled water	140 °C—20 min
HYACQD2	500 µL HYA solution, 0.005 g caffeine, 0.005 g L-cysteine, 0.05 g Na-Borate, 1 mL distilled water	140 °C—20 min
HYACQD3	500 µL HYA solution, 0.005 g caffeine, 0.005 g L-cysteine, 0.025 g Na-Borate, 1 mL distilled water	140 °C—20 min
HYACQD4	500 µL HYA solution, 0.05 g caffeine, 0.005 g L-cysteine, 0.05 g boric acid, 1 mL distilled water	140 °C—20 min
GPCQD	0.2 g GABA-Pentin, 0.005 g L-cysteine, 2 mL distilled water	140 °C—20 min
CitCQD	0.1 g Citicoline, 1 mL distilled water	140 °C—20 min
FCQD	0.01 g Favipiravir, 1 mL distilled water	140 °C—20 min
CRCQD	0.2 g Citrulline, 1 mL distilled water	140 °C—20 min
SDCQD1	0.1 g Sulfadiazine, 0.005 g L-cysteine, 1 mL distilled water	140 °C—20 min
SDCQD2	0.2 g Sulfadiazine, 1 mL distilled water	140 °C—20 min
BRCQD	0.2 g Bromelain, 1 mL distilled water	150 °C—20 min
HTCQD	0.01 g Hydrochlorothiazide, 0.005 g urea, 1 mL distilled water	150 °C—20 min
IMCQD	0.01 g Indomethacin, 0.005 g urea, 1 mL distilled water	150 °C—20 min
PSCQD	0.01 g Paracetamol, 0.005 g urea, 1 mL distilled water	140 °C—20 min
LPCQD1	0.01 g α-Lipoic acid, 0.005 g urea, 1 mL distilled water	150 °C—20 min
LPCQD2	0.01 g α-Lipoic acid, 1 mL distilled water	150 °C—20 min
CafCQD	0.2 g CAMH, 0.1 g caffeine, 1 mL distilled water	180 °C—20 min
MLCQD	0.01 g Mesalazine, 1 mL distilled water	100 °C—20 min

## Data Availability

The data presented in this study are available on request from the corresponding author. The data are not publicly available due to patent considerations and applications.

## Abbreviations

The following abbreviations are used in this manuscript:

APIs	Active pharmaceutical ingredients
BRCQD	Bromelain carbon quantum dot
BupiCQD	Bupivacaine carbon quantum dot
CafCQD	Caffeine carbon quantum dot
CAMH	Citric acid monohydrate
CitCQD	Citicoline carbon quantum dot
CQD	Carbon quantum dots
CRCQD	Citrulline carbon quantum dot
EDA	Ethylenediamine
EnCQD	Enalapril carbon quantum dot
FCQD	Favipiravir carbon quantum dot
GPCQD	GABA-Pentin carbon quantum dot
GQDs	Graphene quantum dots
HTCQD	Hydrochlorothiazide carbon quantum dot
HYACQD	Hyaluronic acid carbon quantum dot
IMCQD	Indomethacin carbon quantum dot
LCQD	Lidocaine carbon quantum dot
LPCQD	α-Lipoic acid carbon quantum dot
MetCQD	Metformin carbon quantum dot
MLCQD	Mesalazine carbon quantum dot
Na-Borat	Sodium borate
PSCQD	Paracetamol carbon quantum dot
q-Drugs	Quantum Drugs
QDs	Quantum dots
SalCQD	Salicylic acid carbon quantum dot
SDCQD	Sulfadiazine carbon quantum dot
SerCQD	Sertraline carbon quantum dot
